# Supplementary material for: Reconciling Mining with the Conservation of Cave Biodiversity: A Quantitative Baseline to Help Establish Conservation Priorities
Source: PLoS One. 2016 Dec 20;11(12):e0168348. doi: 10.1371/journal.pone.0168348 (PMC5173368; doi:10.1371/journal.pone.0168348)
Supplement: S1 Dataset — (ZIP) [file pone.0168348.s002.zip › Taxa/Serra Norte/SN_2013/N4E/Lista N4E-57.pdf]

CAVIDADE N4E-0057

Classe	Ordem	Fam/Outros	Gên/Outros	Espécie	S-U	U-S	Zon.
Arachnida	Acari	(Oribatida)		jovem	X		E
Arachnida	Acari	Acaridae		sp.1		X	E
Arachnida	Acari	Ixodidae	*Amblyomma*	sp.1		X	P
Arachnida	Acari	Prostigmata		sp.49	X		P
Arachnida	Acari	Trombiculidae		sp.39		X	P
Arachnida	Amblypygi	Charinidae	*Charinus*	sp.1		2	E
Arachnida	Amblypygi	Phrynidae	*Heterophrynus*	*longicornis*	2	3	P
Arachnida	Araneae	Ctenidae		jovem	2		P
Arachnida	Araneae	Drymusidae	*Drymusa*	*spelunca*	X		P
Arachnida	Araneae	Ochyroceratidae	*Speocera*	sp.1	X	X	E/P
Arachnida	Araneae	Pholcidae	*Leptopholcus*	sp.1	X		E
Arachnida	Araneae	Pholcidae	*Mesabolivar*	sp.1	X	X	E
Arachnida	Araneae	Salticidae		sp.1	X	X	E
Arachnida	Araneae	Scytodidae	*Scytodes*	*eleonorae*		1	P
Arachnida	Araneae	Theraphosidae	aff. *Holothele*	sp.1		2	E
Arachnida	Araneae	Theridiidae		jovem	X		P
Arachnida	Araneae	Theridiosomatidae	*Plato*	sp.1	X	X	E/P
Arachnida	Opiliones	Stygnidae	*Protimesius*	*gracilis*	1		P
Arachnida	Pseudoscorpiones	Chernetidae	*Spelaeochernes*	sp.1	X	X	E/P
Arachnida	Pseudoscorpiones	Chthoniidae	*Pseudochthonius*	sp.1		X	E/P
Arachnida	Scorpiones	Buthidae	*Ananteris*	jovem	1		E
Chilopoda	Scolopendromorpha	Cryptopidae	*Cryptops*	sp.1		3	E/P
Chilopoda	Scolopendromorpha	Scolopocryptopidae		jovem	2		E
Chilopoda	Scutigeromorpha	Pselliodidae		jovem	1	1	P
Diplopoda	Glomeridesmida	Glomeridesmidae		sp.1		1	P
Diplopoda	Polydesmida	Chelodesmidae		sp.1		1	P
Diplopoda	Polydesmida	Pyrgodesmidae		sp.1		X	E/P
Entognatha	Collembola	Cyphoderidae		sp.1		X	E
Entognatha	Collembola	Isotomidae		sp.1		X	P
Entognatha	Collembola	Neelidae		sp.1		X	E
Entognatha	Collembola	Paronellidae		sp.1	X	X	E/P
Entognatha	Collembola	Paronellidae		sp.4	X	X	E/P
Entognatha	Diplura	Campodeidae		sp.1	X	X	E/P
Insecta	Blattodea	Blattellidae		jovem		1	P
Insecta	Blattodea	Polyphagidae		jovem		1	E
Insecta	Blattodea			jovem	3		P
Insecta	Coleoptera	Carabidae		sp.5	X		E
Insecta	Coleoptera	Coccinellidae		sp.2		X	E
Insecta	Coleoptera	Staphylinidae		sp.41	X		E
Insecta	Diptera	Chironomidae		sp.	X		E
Insecta	Diptera	Phoridae	Phorinae	sp.		X	P
Insecta	Diptera	Psychodidae	Phlebotominae	sp.	X	X	E/P
Insecta	Heteroptera	Reduviidae	Emesinae	sp.6	1	1	E
Insecta	Heteroptera	Reduviidae	Reduviinae	sp.	1	2	E/P
Insecta	Homoptera	Cicadellidae		sp.2		X	E
Insecta	Homoptera	Cixiidae		sp.3	X		E
Insecta	Homoptera	Cixiidae		sp.5		X	P
Insecta	Hymenoptera	Formicidae	*Camponotus*	sp.1	X	X	E/P
Insecta	Hymenoptera	Formicidae	*Camponotus*	sp.2		X	E/P

Insecta	Hymenoptera	Formicidae	*Crematogaster*	sp.1	X		E/P
Insecta	Hymenoptera	Formicidae	*Gnamptogenys*	sp.1	X	X	E/P
Insecta	Hymenoptera	Formicidae	*Gnamptogenys*	sp.4		X	P
Insecta	Hymenoptera	Formicidae	Myrmicinae	sp.1	X		E
Insecta	Hymenoptera	Formicidae	*Pachycondyla*	sp.1	X		P
Insecta	Hymenoptera	Formicidae	*Pachycondyla*	sp.2		X	P
Insecta	Hymenoptera	Formicidae	*Paratrechina*	sp.2	X	X	E/P
Insecta	Hymenoptera	Formicidae	*Solenopsis*	sp.1		X	E
Insecta	Isoptera	Termitidae	*Nasutitermes*	sp.	X		E/P
Insecta	Lepidoptera	Noctuoidea		sp.5	X	2	E
Insecta	Neuroptera	Myrmeleontidae		jovem		X	E
Insecta	Orthoptera	Gryllidae	Nemobiinae	sp.	1	1	P
Insecta	Orthoptera	Phalangopsidae	*Paraclodes*	sp.	1		
Insecta	Orthoptera	Phalangopsidae	*Phalangopsis*	sp.	31	100	E/P
Insecta	Orthoptera	Tettigoniidae	Listroscelidinae	sp.	1		
Insecta	Psocoptera	Epipsocidae	*Epipsocus*	sp.2	X		E/P
Insecta	Psocoptera	Peripsocidae	*Peripsocus*	sp.1	X		E
Insecta	Psocoptera	Psyllipsocidae	*Psyllipsocus*	sp.1		X	E
Gastropoda	Pulmonata	Bulimulidae	*Naesiotus*	sp.	X		P
Gastropoda	Pulmonata	Subulinidae		sp.4		X	P
Gastropoda	Pulmonata	Systrophiidae	*Happia*	sp.	X		P
Malacostraca	Isopoda	Armadillidae		sp.1	X		E/P
Malacostraca	Isopoda	Scleropactidae	*Circoniscus*	*buckupi*		X	P
Symphyla		Scutigerellidae		sp.		X	E/P
Amphibia	Anura	Strabomantidae	*Pristimantis*	cf. *fenestratus*	7		E/P
Mammalia	Chiroptera	Emballonuridae	*Peropteryx*	*kappleri*	3	5	E/P
Mammalia	Chiroptera	Furipteridae	*Furipterus*	*horrens*	3		P
Mammalia	Chiroptera	Phyllostomidae	*Carollia*	*perspicillata*	1	1	P
Mammalia	Chiroptera	Phyllostomidae	*Glossophaga*	*soricina*		1	P
Reptilia	Squamata	Gekkonidae	*Thecadactylus*	sp.	1		E

